# Arbuscular Mycorrhizal Symbiosis Affects Plant Immunity to Viral Infection and Accumulation

**DOI:** 10.3390/v11060534

**Published:** 2019-06-08

**Authors:** Zhipeng Hao, Wei Xie, Baodong Chen

**Affiliations:** 1 State Key Laboratory of Urban and Regional Ecology, Research Center for Eco-Environmental Sciences, Chinese Academy of Sciences, Beijing 100085, China; xieweibisheng@yeah.net (W.X.); bdchen@rcees.ac.cn (B.C.); 2 University of Chinese Academy of Sciences, Beijing 100049, China

**Keywords:** mycorrhiza, immune priming, virus-responsive genes, root colonization, symptom severity

## Abstract

Arbuscular mycorrhizal (AM) fungi, as root symbionts of most terrestrial plants, improve plant growth and fitness. In addition to the improved plant nutritional status, the physiological changes that trigger metabolic changes in the root via AM fungi can also increase the host ability to overcome biotic and abiotic stresses. Plant viruses are one of the important limiting factors for the commercial cultivation of various crops. The effect of AM fungi on viral infection is variable, and considerable attention is focused on shoot virus infection. This review provides an overview of the potential of AM fungi as bioprotection agents against viral diseases and emphasizes the complex nature of plant–fungus–virus interactions. Several mechanisms, including modulated plant tolerance, manipulation of induced systemic resistance (ISR), and altered vector pressure are involved in such interactions. We propose that using “omics” tools will provide detailed insights into the complex mechanisms underlying mycorrhizal-mediated plant immunity.

## 1. Introduction

Arbuscular mycorrhiza (AM), a mutualistic association between the roots of most land plants and fungi from the phylum Glomeromycota [1], confers a series of benefits to host plants [2]. AM fungi improve plant growth and fitness in exchange for carbohydrates from their host to complete their life cycle [3,4]. The mycorrhizal extracellular hyphal spreads widely into the soil, thereby acquiring water and nutrients, especially phosphate [5]; AM fungus also develops in roots where the AM fungus colonizes the cortex and forms highly branched intracellular structures called arbuscules, which facilitate the transfer of mineral nutrients to the root cells. In addition to the improved plant nutritional status, the physiological changes that trigger metabolic changes in the root via AM fungi can also increase the host ability to overcome biotic and abiotic stresses [6,7,8,9,10]. A mycorrhizal bioprotection effect has been observed against soil-borne fungal pathogens that cause wilting or root rot [11,12], and AM symbiosis induces host plant resistance against below-ground and shoot pathogens, nematodes, or chewing insects [13,14,15,16]. In this review, we will focus on the contribution of AM fungi in plant and virus interactions, which have been less investigated.

Viral diseases in plants are a major threat to food security worldwide, and this problem is exacerbated by crop management practice and climate changes [17]. The control of plant viruses is mainly based on prevention by using genetically resistant plants and through vector eradication [18]. However, resistance sources are lacking in many cases, and genetic resistance achieved by genetic engineering can be overcome by viruses as it is usually based on a gene-for-gene interaction [19]. Common strategies to control plant virus infection that target vectors via agrochemicals are unacceptable because of their high cost and potential adverse environmental effects. The possibility of priming plant immunity against viruses by using beneficial microorganisms, such as AM fungi, deserves considerable interest. Therefore, this review aims to provide an overview of the impact of AM symbiosis on viral pathogen infection and the mechanisms involved in such interactions.

## 2. Impact of AM Symbiosis on Viral Development

The studies related to the interactions between AM symbiosis and viral pathogens are summarized in Table 1. The effect of AM fungi on viral infection is variable, and considerable attention is focused on shoot virus infection. Focusing on disease severity, Shaul et al. [20] analyzed the interactions between *Rhizophagus intraradices* and *Tobacco mosaic virus* (TMV) in tobacco leaves and observed that the disease symptoms are more enhanced in mycorrhizal plants than in non-mycorrhizal plants. Similarly, Nemec and Myhre [21] demonstrated that the leaf shock symptom development is severe in mycorrhizal *Citrus* rootstocks. The increase in virus accumulation in leaves of *Potato virus Y*-infected mycorrhizal strawberry and potato plants was observed [22,23]. Mycorrhizal plants could become increasingly sensitive to viral presence over time as virus concentration continuously increases compared with that in non-mycorrhizal controls [24]. During the early stages, decreased or no difference in symptom severity or virus infection is observed in mycorrhizal plants compared with non-mycorrhizal plants [22]. A study demonstrates a clearly protective effect of AM fungi against viral infections in roots and shoots and in disease symptoms [25]. Recently, Hao et al. [26] showed that mycorrhizal colonization significantly decreases nematode vector *Xiphinema index* reproduction in soil and gall formation on roots and protects grapevine against grapevine fanleaf virus (GFLV). These inconsistent findings related to mycorrhizal plants and viral pathogens that vary with the plant–AM fungus–virus interactions involved have been reported. 

### 2.1. AM Fungi

The reviewed experiments are limited to the single inoculation of one of the following species: *Funneliformis mosseae*, *Funneliformis geosporum*, *Rhizophagus intraradices*, and *Glomus* sp. (Table 1). These species can be easily propagated and are the most common symbionts which were geographically distributed at a global scale [27]. Monospecies inoculum is used in all these studies. Berruti et al. [3] used a meta-analysis to show that plant growth promotion effects are more successful in single-species mycorrhizal inoculation experiments than in experiments with more than one AM fungi species. This phenomenon can be explained by the fact that the functional redundancy in AM fungi resulted in few fungal species that can alleviate stress and benefit a plant [28]. Furthermore, different isolates within the same AM fungi species can increase the variations in plant response [29], suggesting that functional heterogeneity exists in these species. Though the current general trend that uses single species in these reviewed studies is reasonable, the selection of remarkably effective AM fungi strains for AM fungus–virus interactions is also required.

### 2.2. Viruses

Virus identity appears to play an important role in the impact of AM symbiosis on virus infections given that viral infection of potato plants resulted in a variety of symptoms depending on the viral strain. The interactions between AM fungi and ssRNA(+) viral plant pathogens, the single largest group of RNA viruses, have been extensively studied. These studies cover 6 out of 30 families in this group of RNA viruses. Among ssRNA(+) viruses, including Bromoviridae, Closteroviridae, Potyviridae, Secoviridae, and Virgaviridae. Only one strain of the Bromoviridae family shows an insignificant difference in cucumber mosaic virus (CMV-Y, yellow strain) accumulation in the cucumber leaves of mycorrhizal and control cucumber plant determined using indirect enzyme-linked immunosorbent assay (ELISA) at 1, 2 and 3 weeks after inoculation [30]. Once inside the plant, this virus can inhibit the plant’s ability to signal for gene silencing in other tissues, thereby furthering CMV-Y infection. Similar to other viruses, this kind of virus replicates in the cytoplasm, and moves through the plasmodesmata via cell-to-cell transfers, but the phloem can be utilized for long-distance movement in the plant. Other studies demonstrate a negative effect of AM fungi against ssRNA(+) virus in the shoot and root, leading to an increased virus concentration or symptom severity in mycorrhizal plants.

No change in virus accumulation or symptom severity is observed between mycorrhizal and non-mycorrhizal plants 14 days post-virus inoculation (dpi) of tomato spotted wilt virus (TSWV), an RNA virus of ambisense polarity belonging to the family Peribunyaviridae. However, a prolonged increase in virus titer is observed in mycorrhizal plants in a longer period [24]. “Recovery” is a phenomenon defined by the reduction or disappearance of symptoms in virus-infected plants that initially exhibit severe disease and by being protected from reinoculation with the same virus [31]. Results show that 25% of mycorrhizal plants and 65% of non-mycorrhizal plants recovered at 34 dpi, and the decreased of recovery in mycorrhizal plants indicates that the plant’s response to TSWV infection is attenuated by mycorrhizal colonization [24].

Mycorrhizal colonization has a beneficial effect in attenuating the disease caused by tomato yellow leaf curl Sardinia virus (TYLCSV), an ssDNA virus belonging to the Geminiviridae family, and causes one of the most serious viral diseases of tomatoes [25]. Considering that geminiviruses colonize the nucleus of cells and are phloem-limited [32], these studies suggest that the different results observed with virus is likely due to the particular nature of the viruses. 

### 2.3. Plants

A plant is another related aspect that should be considered. Nine plant species are reviewed, while, four studies focus on tomato, including different varieties with a range of genetic differences (Table 1). AM fungi protect the same variety of tomato (“Moneymaker”) against the TYLCSV, but not from the TSWV [24,25]. Different plant species show a significantly different responsiveness to mycorrhizal inoculation [5,33], and the effectiveness of mycorrhizal bioprotection depends on the plant species involved. Though major crops, such as wheat and maize, have relatively high mycorrhizal dependence on plant growth and nutrient uptake, negligible attention has been given to identifying the bioprotection effects against viral disease on these plants.

### 2.4. Environment Conditions

Many factors also affect the success of bioprotection, including AM fungi species compatibility with the target environment and degree of competition with other soil organisms in the timing of inoculation. All experiments are conducted under controlled (greenhouses) conditions (Table 1), and pots are usually filled with sterilized substrates with low mineral nutrients, leading to optimized requirements for viruses and AM fungi to infect or colonize compared with that in open-field conditions. Given that high nutrient levels in substrates can reduce AM fungi colonization [5], virus infection is difficult in highly sophisticated management cultivation measures [18]. The positive or negative impacts of AM fungi on plant–virus interactions can be eased or reversed via agricultural practices and in multiple stressed environmental conditions. Additional insights into the variability in tripartite performance in a range of different environments can help increase the efficacy of AM fungi.

## 3. Mechanisms of Interactions between AM Fungi and Plant Viruses

**Figure 1:**
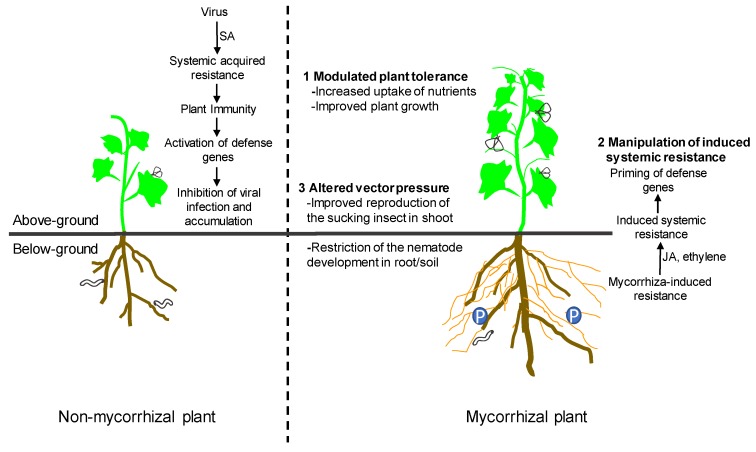
The underlying mechanisms of the impact of arbuscular mycorrhizal (AM) symbiosis on infection by viral pathogens, including modulated plant tolerance, the manipulation of induced systemic resistance, and altered vector pressure. SA, salicylic acid; JA, jasmonic acid.

The underlying mechanisms of the impact of AM symbiosis on infection by viral pathogens remain poorly understood. Several mechanisms, including modulated plant tolerance, the manipulation of induced systemic resistance (ISR), and altered vector pressure are involved in such interactions (Figure 1). Mycorrhizal effects likely result from a combination of several mechanisms [13,34]. The mechanisms responsible for the specific plant–AM fungus–virus interaction highlight the need to consider mycorrhizal symbiosis in the context of plant immunity to exploit potential benefits for plant bioprotection.

### 3.1. Modulated Plant Tolerance

Mycorrhizal hyphae are considerably thinner than roots and can penetrate smaller pores; they emerge from the root surface and acquire macro and micronutrients from soil volumes that are inaccessible to roots [35]. The increased uptake of nutrients in the host plant may affect the susceptibility of the plant to viral infection. Maffei et al. [25] showed that TYLCSV-infected mycorrhizal plants exhibit less severe symptoms than infected non-mycorrhizal plants, but non-mycorrhizal plants do not suffer from phosphate starvation when they are watered with a modified nutrient solution with optimized phosphate content. This study indicates that the improved nutritional status of mycorrhizal plant alone could not explain its bioprotection against viruses. Daft and Okusanya [22] demonstrated that enhanced virus production can be achieved in non-mycorrhizal plants under higher phosphate levels, and the amount of Arabis mosaic virus from strawberry plants is greater in mycorrhizal than in non-mycorrhizal plants, thus, these results could be attributed to the high phosphate levels in mycorrhizal plants. Borer et al. [36] also showed that increased phosphorous content is associated with an increase in barley and cereal yellow dwarf virus infection. Thus, AM fungi can benefit virus development rather than provide bioprotection. Given the high-affinity phosphate transporter in an AM fungus [37], the nutritional aspects of AM symbiosis have been studied extensively from the molecular perspective [38]. The AM marker gene *LePT4*, a mycorrhiza-specific phosphate transporter, is preferentially expressed in arbuscule-containing cells of mycorrhizal tomato roots [39,40]. However, the expression profiles of *LePT4* in mycorrhizal plants are not modified by TYLCSV infection [25].

Furthermore, the improved nutrient status of mycorrhizal plants, especially under nutrient deficient conditions, leads to vigorous plant growth, which can compensate for viral damage. However, improved plant growth may impact mycorrhizal plants as it increases potential virus multiplication [21]. Though TSWV infection significantly suppresses the AM-induced growth increase, mycorrhizal tomato plants showed a growth increase compared with that of mock-inoculated ones under viral stress [24]. Therefore, enhanced growth in mycorrhizal plants could also compensate for damage caused by viruses, and mycorrhizal symbiosis still benefits plants because they are able to tolerate increased viral pressure under certain conditions. 

### 3.2. Manipulation of Induced Systemic Resistance

The modulation of plant physiology and signal transduction pathways during mycorrhizal symbiosis formation and function has received considerable attention [41,42,43]. A transient and weak activation of plant immune system takes place in response to mycorrhizal colonization, with the elicitation of specific defense reactions [44]. Microbe-associated molecular patterns (MAMPs) from AM fungi are recognized by the innate immune system of a host plant, while MAMP-triggered immunity (MTI) could prime the salicylic acid-dependent defense responses [45]. The enhanced production of phytoalexins and phenolic compounds; accumulation of hydrolytic enzymes, such as chitinases and β-1,3-glucanases; and activation of phenylpropanoid metabolism in plant roots are involved in MTI on AM fungal colonization [34]. The pre-conditioning of the host plant elicited by AM fungi strengthens and speeds up systemic defense responses against subsequent plant pathogens [12]. Phytoalexin synthesis and cell wall fortification, which are effective against bacterial or fungal pathogens [46], usually could not prevent virus spread or replication [47]. β-1,3-glucanases, which inhibit callose deposition degradation in plasmodesmata, could be accumulated in mycorrhizal cucumber plants [48], and delay cell-to-cell virus spread and loading into phloem [49]. Such priming of plant defense conferred by mycorrhizal symbiosis may be involved in AM fungi-mediated bioprotection against plant viruses.

Mycorrhiza-induced resistance (MIR) against many plant fungal and bacterial pathogens shares common characteristics with the systemic acquired resistance (SAR) after pathogen attack and is associated with the SAR-like priming of defense response, such as the accumulation of pathogenesis-related (PR) proteins [34]. Gallou et al. [50] observed a strong induction of PR protein genes (*PR1* and *PR2*) in mycorrhizal plants challenged with *Phytophthora infestans* in vitro. However, the accumulation and mRNA steady-state levels of PR proteins, PR-1 and PR-3, are low in the leaves of mycorrhizal plants infected with the tobacco mosaic virus [20]. The expression of PR proteins is tightly correlated with the SA signal transduction pathway during necrotic lesion formation in tobacco–virus interactions [51]. SA levels are enhanced in TSWV-infected mycorrhizal and non-mycorrhizal plants [24], while Shaul et al. [20] further indicated that the delayed *PR-1* and *PR-3* gene expression in mycorrhizal plants infected by TMV is not involved in SA-dependent defense, because the exogenous application of SA to the foliage does not abolish the mycorrhizal plant response. 

The priming of jasmonic acid (JA)-dependent defense responses is demonstrated in mycorrhizal plants under a pathogen infection unlike in the non-mycorrhizal control [52]. AM fungi reduce the development of plant pathogens through ISR, a resistance phenomenon usually induced by non-pathogenic microorganisms [10]. Reports of decreased pathogen development in shoot or in non-mycorrhizal parts of mycorrhizal root systems using a split-root system is confirmed as a mycorrhizal-mediated ISR [53,54,55].

ISR is predominantly regulated by the JA-mediated and ethylene-mediated signaling pathways [34]. Li et al. [56] showed that mycorrhizal plants have a higher JA content compared with that of non-mycorrhizal plants during *Phytophthora sojae* infection, and Pozo et al. [57] demonstrated that the expression of marker genes for JA responses are significantly increased in mycorrhizal tomato plants. JA has a limited effect in early local defenses of potatoes infected with potato virus Y-NTN [58], and Moizzi et al. [24] indicated that increased levels of JA in mycorrhizal plants are detected with or without TSWV infection. Considering that the SA and JA pathways are usually mutually antagonistic, these signaling pathways may not act independently but influence each other through a complex network in virus–AM fungus–plant interactions [59].

A high-throughput transcriptional profiling analysis via microarrays is conducted to monitor transcriptional changes in the roots and shoots of mycorrhizal plant infection with TSWV, and this transcriptome study may shed light on tripartite interaction [24]. The number of differentially expressed (DE) genes in the roots of TSWV-infected mycorrhizal plants is higher compared with that measured in the single-inoculation treatments. In shoots, the impact of combined TSWV and AM fungus appears intermediate between that observed for the mycorrhizal (lowest) and the virus (highest) interaction separately. A total of 215 genes modified the regulation in the shoots TSWV-infected mycorrhizal plants, while 579 DE genes were found in the roots. This transcriptome data indicates that the expression levels of several candidate virus-responsive upregulated genes related to sugar metabolism, defense, and response to hormones are reduced in mycorrhizal plants compared with that in non-mycorrhizal plants after TSWV infection. For example, the expression of PR protein 10, which has antimicrobial activity, is downregulated in TSWV-infected mycorrhizal plants, but PR protein 10 could be linked to the reduction of plum pox virus infection in *Nicotiana tabacum* [60]. On the basis of the suppression subtractive hybridization study, Hao et al. [55] showed that glutathione S-transferase (GST) is upregulated during MIR. GST is involved in the detoxification of reactive oxygen species and is upregulated in response to TSWV [61] but is not activated in virus-infected mycorrhizal plants [24]. However, only a few transcriptomes of AM fungi-associated changes are available, and future “omics” studies of viral attackers might clarify whether the AM fungi priming of plant defenses is effective.

### 3.3. Altered Vector Pressure

The control of virus diseases could also be based on prevention by eradication of insect, nematode and fungal vectors [18]. These mycorrhizal protective effects range from enhanced plant tolerance to a reduction in pathogen infection [14,16,62]; therefore, the potential of AM symbiosis to restrict these vectors may contribute to diminishing viral disease severity. The soil-borne GFLV spreads mainly via the nematode vector *X. index*, which is suppressed by the AM fungus *R. intraradices* with induced local and systemic protection [55]. Therefore, the bioprotection effects of AM symbiosis to restrict a vector to biologically realistic thresholds may limit GFLV infection. The reduced viruliferous nematode development after *R. intraradices* inoculation does not exclude GFLV infection at an extremely high nematode pressure (100 nematodes per plant), but GFLV is absent from mycorrhizal grapevine roots 90 days after nematode inoculation at a low nematode pressure (10 nematodes per plant, approximately the levels of nematode abundance observed in vineyards [63,64,65]) but detectable in non-mycorrhizal roots [26]. Thus, management of the nematode vector by using AM fungi has a potential to diminish GFLV disease severity. 

However, the colonization of *Plantago lanceolata* by AM fungi improves the growth and reproduction in the shoot of the sucking insect *Myzus persicae* [66,67], which acts as a vector for the transport of various mosaic viruses, potato leafroll virus, potato virus Y, and Mikania micrantha wilt virus [68]. As a virus depends on a vector for its survival and transmission, the improved performance of this aphid on mycorrhizal plants may lead to enhanced plant infection by the viruses. 

For fungal vectors, root colonization by AM fungi is associated with symptomless root parasites, *Olpidium* species [69,70], which are also known as the vectors of viruses on cereals, tobacco and salad [71,72]. The potential of AM fungi to reduce fungal pathogen infections has been shown frequently [16,34], but no information is available concerning mycorrhizal protection against fungal vectors mediating virus transmission. 

## 4. Conclusions and Future Directions

We provide an overview of the potential of AM fungi as bioprotection agents against viral diseases and emphasize the complex nature of plant–fungus–virus interactions. However, data are still limited to certain stages of virus symptoms, and the actual long-term processes attained by inoculating plants with AM fungi must be evaluated case-by-case in the field. These interactions depend on several biotic and abiotic factors, and practices such as the use of pesticides or fertilization (especially that of phosphorous), can be controversial for plant–fungus–virus interactions. The technological progress unraveled the mechanisms proposed for mycorrhizal-mediated bioprotection, and relevant strategies, such as next-generation sequencing, may further elucidate the mechanisms of induced resistance of AM symbiosis. The complex relationship between the systemic priming of plant defenses and the suppression of immunity, which are required for the establishment of AM symbiosis, must also be further studied involving “omics” tools [73].

In addition, the bioprotection efficiency of AM fungi may be improved if they are used in combination with other biological control agents. Elsharkawy et al. [30] showed that the co-inoculation of cucumber plants with AM fungi and a plant growth-promoting fungus *Fusarium equiseti* results in the effective control of CMV development, and the importance of microorganisms in rhizosphere and phyllosphere will be confirmed with microbiome studies. Though most studies on plant–AM fungus–virus interactions have been conducted under controlled conditions, the development of mycorrhizal inocula for large-scale field application is growing quickly [14,74]. In view of sustainable agriculture, unveiling the principles behind the functional interplay among the tripartite will be of major interest to the effective application of AM fungi in an integrated viral management program.

## Figures and Tables

**Table 1:** Table summarizing the studies related to the interactions between arbuscular mycorrhizal (AM) symbiosis and viral pathogens.

	Virus				AM Fungus	Plant	Mycorrhizal Effects on Virus Development	References
Code	Group	Family	Genus	Species				
1	Group II: ssDNA	Geminiviridae	*Begomovirus*	Tomato yellow leaf curl Sardinia virus	*Funneliformis mosseae* (Syn. *Glomus mosseae* BEG12)	Tomato (*Solanum lycopersicum* L. (‘Moneymaker’))	Both root and shoot concentrations of viral DNA are lower in mycorrhizal plants than in control plants.	[25]
2	Group IV: ssRNA(+)	Bromoviridae	*Ilarvirus*	Citrus leaf rugose virus (CLRV-2)	*Funneliformis geosporum* (Syn. *Endogone macrocarpa* var. Geospora)	Citrus rootstocks (alemow (*Citrus macrophylla* Wester); grapefruit (*Citrus paradisi* Macf. ‘Duncan’); sour orange (*Citrus aurantium* L.))	The leaf shock symptom development is more severe in mycorrhizal plants than in control plants.	[21]
3	Group IV: ssRNA(+)	Bromoviridae	*Cucumovirus*	Cucumber mosaic virus (CMV-Y, yellow strain)	*Funneliformis mosseae* (Syn. *Glomus mosseae*)	Cucumber (*Cucumis sativus* L. cv. Tokiwa Jibai)	No significant difference is observed between mycorrhizal and control treatments.	[30]
4	Group IV: ssRNA(+)	Closteroviridae	*Closterovirus*	Citrus tristeza virus (a severe tristeza isolate, T-3; a severe tristeza isolate, T-3)	*Claroideoglomus etunicatum* (Syn. *Glomus etunicatum*)	Citrus rootstocks (alemow (*Citrus macrophylla* Wester); grapefruit (*Citrus paradisi* Macf. ‘Duncan’); sour orange (*Citrus aurantium* L.))	Virus-induced root degeneration is observed in both control and mycorrhizal plants.	[21]
5	Group IV: ssRNA(+)	Potyviridae	*Potyvirus*	Potato virus Y	*Funneliformis geosporum* (Syn. *Endogone macrocarpa* var. Geospora)	Strawberry (*Fragaria × ananassa* Duch. var. Talisman)	Virus contents of the mycorrhizal plants are higher than those of corresponding control plants.	[22]
6	Group IV: ssRNA(+)	Potyviridae	*Potyvirus*	Potato virus Y	*Rhizophagus intraradices* (Syn. *Glomus intraradices* isolate no. OM/95)	Potato (*Solanum tuberosum* L. cv. ‘Marfona’)	Reproduction of the virus and disease severity are significantly increased in mycorrhizal plants than in control plants.	[23]
7	Group IV: ssRNA(+)	Secoviridae	*Nepovirus*	Arabis mosaic virus	*Funneliformis geosporum* (Syn. *Endogone macrocarpa* var. Geospora)	Petunia (*Petunia hybrida* (J. D. Hooker) Vilmorin, var. Rose of Heaven),	Both the leaves and roots of the mycorrhizal plants contain more virus at each harvest than those of control plants.	[22]
8	Group IV: ssRNA(+)	Secoviridae	*Nepovirus*	Grapevine fanleaf virus	*Rhizophagus intraradices* (Syn. *Glomus intraradices* isolate BEG141)	Grapevine rootstock (*Vitis berlandieri×Vitis riparia* SO4)	The virus is present in both non-mycorrhizal and mycorrhizal plants at a high abundance of the nematode vector, while, the virus is detected only in non-mycorrhizal roots but absent from mycorrhizal grapevine at a low vector abundance.	[26]
9	Group IV: ssRNA(+)	Virgaviridae	*Tobamovirus*	Tomato (aucuba) mosaic virus	*Funneliformis geosporum* (Syn. *Endogone macrocarpa* var. Geospora)	Tomato (*Lycopersicon esculentum* Mill. F1 hybrid, var. Eurocross A)	Control leaves contain more virus at early stage (4 and·7 days), while the rate of virus multiplication was faster in the leaves of mycorrhizal plants, which leaded to more virus accumulation in long-term (14 and 21 days).	[22]
10	Group IV: ssRNA(+)	Virgaviridae	*Tobamovirus*	Tobacco mosaic virus (strain U1)	*Rhizophagus intraradices* (Syn. *Glomus intraradices* isolate no. OM/95)	Tobacco (*Nicotiana tabacum* cv. Xanthi-nc)	Leaves of mycorrhizal plants show a higher severity of symptoms than those of control plants.	[20]
11	Group V: ssRNA(-)	Peribunyaviridae	*Tospovirus*	Tomato spotted wilt virus (isolate T1012)	*Funneliformis mosseae* (Syn. *Glomus mosseae* isolate BEG12)	Tomato (*Solanum lycopersicum* L. (‘Moneymaker’))	No differences in symptom severity or virus concentration are observed between mycorrhizal and non-mycorrhizal plants in short time (14 days post-virus inoculation (dpi)), while an increase in virus titer is detected in mycorrhizal plants in a longer period (34 and 56 dpi).	[24]
